# Orthologous proteins of experimental de- and remyelination are differentially regulated in the CSF proteome of multiple sclerosis subtypes

**DOI:** 10.1371/journal.pone.0202530

**Published:** 2018-08-16

**Authors:** Nellie A. Martin, Arkadiusz Nawrocki, Viktor Molnar, Maria L. Elkjaer, Eva K. Thygesen, Miklos Palkovits, Peter Acs, Tobias Sejbaek, Helle H. Nielsen, Zoltan Hegedus, Finn Sellebjerg, Tihamer Molnar, Eudes G. V. Barbosa, Nicolas Alcaraz, Ferenc Gallyas, Asa F. Svenningsen, Jan Baumbach, Hans Lassmann, Martin R. Larsen, Zsolt Illes

**Affiliations:** 1 Department of Neurology, Odense University Hospital, Odense, Denmark; 2 Department of Biochemistry and Molecular Biology, University of Southern Denmark, Odense, Denmark; 3 Department of Genetics, Cell- and Immunobiology, Semmelweis University, Budapest, Hungary; 4 Laboratory of Neuromorphology and Human Brain Tissue Bank/Microdissection Laboratory, Semmelweis University, Budapest, Hungary; 5 Department of Neurology, University of Pecs, Pecs, Hungary; 6 Laboratory of Bioinformatics, Biological Research Centre, Szeged, Hungary; 7 Danish Multiple Sclerosis Center, Department of Neurology, Rigshospitalet, University of Copenhagen, Copenhagen, Denmark; 8 Department of Anaesthesiology and Intensive Therapy, University of Pecs, Pecs, Hungary; 9 Computational Biology Group, Department of Mathematics and Computer Science, University of Southern Denmark, Odense, Denmark; 10 The Bioinformatics Centre, Department of Biology, University of Copenhagen, Copenhagen, Denmark; 11 Department of Biochemistry and Medical Chemistry, University of Pecs, Pecs, Hungary; 12 Szentagothai Research Centre, University of Pécs, Pécs, Hungary; 13 Nuclear-Mitochondrial Interactions Research Group, Hungarian Academy of Sciences, Budapest, Hungary; 14 Department of Neurobiology Research, Institute for Molecular Medicine, University of Southern Denmark, Odense, Denmark; 15 Center for Brain Research, Medical University of Vienna, Vienna, Austria; 16 Department of Clinical Research, BRIDGE, University of Southern Denmark, Odense, Denmark; University of Pécs Medical School, HUNGARY

## Abstract

**Objective:**

Here, we applied a multi-omics approach (i) to examine molecular pathways related to de- and remyelination in multiple sclerosis (MS) lesions; and (ii) to translate these findings to the CSF proteome in order to identify molecules that are differentially expressed among MS subtypes.

**Methods:**

To relate differentially expressed genes in MS lesions to de- and remyelination, we compared transcriptome of MS lesions to transcriptome of cuprizone (CPZ)-induced de- and remyelination. Protein products of the overlapping orthologous genes were measured within the CSF by quantitative proteomics, parallel reaction monitoring (PRM). Differentially regulated proteins were correlated with molecular markers of inflammation by using MesoScale multiplex immunoassay. Expression kinetics of differentially regulated orthologous genes and proteins were examined in the CPZ model.

**Results:**

In the demyelinated and remyelinated corpus callosum, we detected 1239 differentially expressed genes; 91 orthologues were also differentially expressed in MS lesions. Pathway analysis of these orthologues suggested that the TYROBP (DAP12)-TREM2 pathway, TNF-receptor 1, CYBA and the proteasome subunit PSMB9 were related to de- and remyelination. We designed 129 peptides representing 51 orthologous proteins, measured them by PRM in 97 individual CSF, and compared their levels between relapsing (n = 40) and progressive MS (n = 57). Four proteins were differentially regulated among relapsing and progressive MS: tyrosine protein kinase receptor UFO (UFO), TIMP-1, apolipoprotein C-II (APOC2), and beta-2-microglobulin (B2M). The orthologous genes/proteins in the mouse brain peaked during acute remyelination. UFO, TIMP-1 and B2M levels correlated inversely with inflammation in the CSF (IL-6, MCP-1/CCL2, TARC/CCL17). APOC2 showed positive correlation with IL-2, IL-16 and eotaxin-3/CCL26.

**Conclusions:**

Pathology-based multi-omics identified four CSF markers that were differentially expressed in MS subtypes. Upregulated TIMP-1, UFO and B2M orthologues in relapsing MS were associated with reduced inflammation and reflected reparatory processes, in contrast to the upregulated orthologue APOC2 in progressive MS that reflected changes in lipid metabolism associated with increased inflammation.

## Introduction

Early in the course of multiple sclerosis (MS), disruption of the blood-brain barrier (BBB) and focal inflammatory demyelination result in acute clinical symptoms followed by recovery (relapsing-remitting, RRMS) [1]. A cascade of events leads to diffuse axonal and neuronal degeneration, and if untreated, RRMS converts into a secondary progressive phase (SPMS) in about 50% of patients; the disease has a progressive course from the onset (primary progressive, PPMS) in about 10–20% of patients [2–4]. A period of diagnostic uncertainty regarding the transition from RRMS to SPMS lasts for several years, and immunomodulatory treatments are less efficacious in progressive disease [5,6]. Biomarkers are needed that predict the natural course and select patients with demand of early highly efficacious treatments.

Cuprizone (CPZ) is a copper chelator, which induces demyelination and oligodendrocyte loss in the corpus callosum and superior cerebellar peduncles of C57BL/6 mice. Suspension of CPZ treatment results in rapid remyelination [7–9]. Some of the mechanisms of oligodendrocyte death overlap with those observed in dying oligodendrocytes of MS lesions [9,10]. Adaptive immune responses are absent or minor in the CPZ model, and the blood-brain barrier (BBB) remains intact in contrast to the inflammatory model of MS, experimental autoimmune encephalomyelitis (EAE) [11].

Omics approaches are promising holistic tools for revealing molecular pathways and quantifying differentially expressed molecules [12]. Focused, hypothesis-driven exploratory translational omics are promising players in omics-driven precision medicine [13]. The cerebrospinal fluid (CSF) is the most proximal body fluid to the central nervous system (CNS), and has been used for biomarker discovery by proteomics in a number of neurological diseases [14].

Here, we hypothesized that combination of omics may focus attention on the importance of certain pathways and molecules in the pathogenesis of multiple sclerosis, and this way we may also identify molecules that are differentially regulated in the CSF of relapsing versus progressive MS. First, we established the transcriptome of experimental de- and remyelination in the CPZ model, and compared the differentially expressed genes to MS lesion transcriptomes in order to identify cellular pathways associated with de- and remyelination in MS. Protein products of overlapping orthologous genes were then screened in the CSF proteome of MS patients, and compared by targeted quantitative proteomics between relapsing and progressive MS. The differentially regulated proteins were correlated with markers of inflammation in the CSF of patients with RRMS, and the expression kinetics of orthologous genes and proteins were examined during demyelination and remyelination in the CPZ model.

## Materials and methods

### Mice

Wild-type C57BL/6 mice were obtained from Taconic Ltd. (Ry, Denmark). Mice were bred at the Biomedical Laboratory, SDU according to protocols and guidelines approved by the Danish Animal Health Care Committee (2014-15-00369). All animal experiments complied with the EU Directive 2010/63/EU for animal experiments, and the study has received approval by the Institutional Animal Care and Use Committee of the University of Southern Denmark, Odense, Denmark. Male mice aged 7–8 weeks were included in the experiments.

### Patients

**Fig 1:**
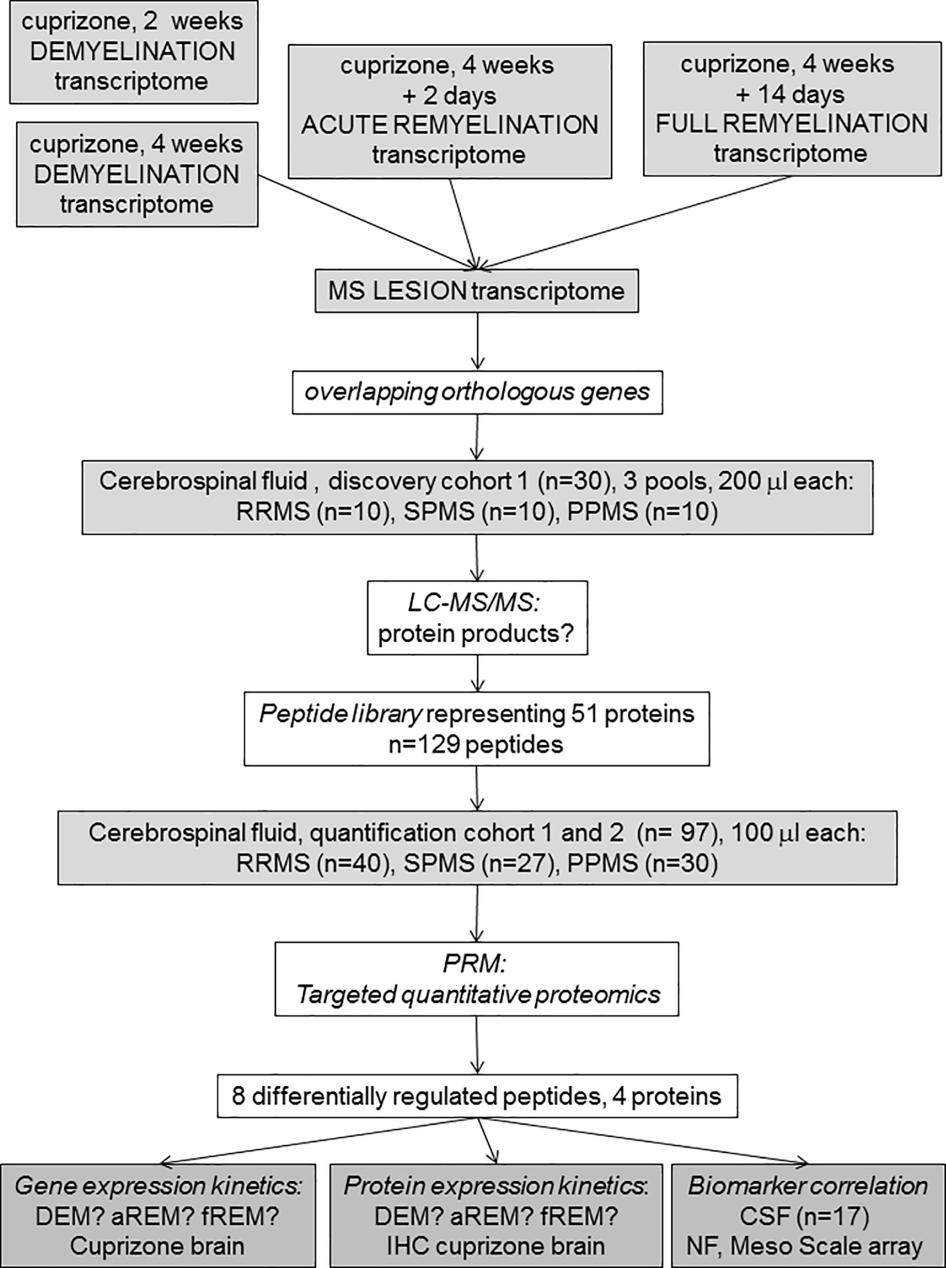
Outline of the experimental design. The transcriptome of punched out corpus callosum was examined at 4 time points in the cuprizone (CPZ) model: early and full demyelination (2 and 4 week CPZ, DEM), acute remyelination (aREM, 2 days after suspending CPZ) and full remyelination (fREM, 14 days after suspending CPZ) by 4x44K Agilent Whole Mouse Genom Expression Microarray. Differentially expressed genes at 4 week demyelination, acute and full remyelination were then compared to published transcriptomes of MS lesions. The CSF proteome was established by liquid chromatography mass spectrometry (LC-MS/MS) in a discovery MS cohort (cohort 1, altogether 30 samples). Protein products of overlapping orthologous CPZ/MS genes were then screened in this MS-CSF proteome. Next, levels of selected 129 peptides of 51 proteins of orthologous CPZ/MS genes were measured in altogether 97 individual MS samples (cohort 1 and 2) by targeted proteomics (parallel reaction monitoring, PRM), and stratified according to relapsing and progressive MS subtypes. The differentially regulated proteins were correlated with concentration of molecular markers of inflammation and axonal degeneration (neurofilament light chain, NFL) [15] in the MS-CSF. The expression kinetics of orthologous genes and proteins was examined by immunohistochemistry (IHC) in the CPZ model and related to demyelination, acute remyelination and full remyelination.

CSF samples were obtained from 30 patients with PPMS (mean age 49±8.6, 17 females), 27 patients with SPMS (45.9±5.8, 14 females), and 40 patients with RRMS (mean age 33.6±10 years, 31 females). Ten subjects of each MS group were used as a discovery cohort for full proteomics of the CSF (cohort 1, altogether 30 MS). In the discovery phase, 10 subjects without neurological disease with diagnostic CSF examination due to headache were also included (mean age 37.7±12.8 years, 7 females). All individual samples from the total MS cohort (cohort 1 and 2, altogether 97 MS samples) were used for quantification of selected peptides by targeted proteomics in relapsing and progressive MS subtypes (**Fig 1**). The CSF was immediately centrifuged at 400 x g for 10 minutes, supernatant was collected and stored at -80°C. The study was conducted in accordance with the approval of the Danish National Ethics Committee (S-20120066) and informed consent was obtained from each subject.

### CPZ-induced demyelination and remyelination

To induce demyelination, powdered standard chow was mixed with 0.2% CPZ (Sigma Aldrich, MO, USA) and administrated for 2 and 4 weeks. Remyelination was examined after 2 days (acute remyelination) or 2 weeks (full remyelination) of regular diet following 4-weeks CPZ feeding. Mice untreated with CPZ were used as controls, and their age and sex were matched to the 4-week demyelination group. Experiments were terminated by euthanizing mice with an overdose of pentobarbital (Glostrup Apotek, Glostrup, Denmark) followed by perfusion with 4% paraformaldehyde (PFA) for staining applications, or phosphate buffered saline for all other applications.

### RNA extraction from the corpus callosum

For the removal of the corpus callosum, the brains were removed from the skull, immediately frozen and cut into coronal serial sections (section thickness 200 μm). By using a stereomicroscope, the corpus callosum was cut out of the sections with a fine Graefe-knife, along its rostro-caudal extension, the samples were collected in ice-cold Eppendorf-tubes and stored frozen until used. RNA was extracted by the miRNeasy micro Kit (Qiagen, Valencia, CA). The quantity and quality of total RNA was assessed by NanoDrop ND-1000 spectrophotometer (NanoDrop Technologies, Wilmington, DE) and Agilent 2100 Bioanalyzer (Agilent Technologies, Palo Alto, CA), respectively. Only those samples that gave >8.0 for RNA integrity number, showed a clear gel image and no DNA contamination was observed on the histogram were used for microarray experiments.

### Microarray

For RNA profiling in untreated controls, during demyelination, acute and full remyelination in the CC, the Agilent Whole Mouse Genom Expression Microarray Kit (G4122F, 4x44K) was applied according to the manufacturer’s instruction (version 1.0) with 100 ng quality-checked total RNA: 3–6 mice were used for each condition. The labeled samples were hybridized for 20 hours at 55°C. The arrays were scanned with an Agilent DNA Microarray Scanner BA, the signal quantification was carried out by Feature Extraction 10.7 Image Analysis Software and data were further analyzed by Genespring GX10.0.

The microarray data are deposited in NCBI Gene Expression Omnibus with accession GSE100663.

### Meso scale discovery electrochemiluminescent assay

Biomarker levels in the CSF were measured by the Meso Scale Discovery (MDS) technique (Mesoscale Discovery, Rockville, USA): Human Proinflammatory Panel 1 V-Plex (IFNγ, IL-1β, IL-2, IL-4, IL-6, IL-8, IL-12p70, TNFα); Human Cytokine Panel 1 V-Plex (GM-CSF, IL-1α, IL-5, IL-7, IL-23/IL-12p40, IL-15, IL-16, IL-17α, TNFβ, VEGF); Human Angiogenesis Panel 1 V-Plex (VEGF-C, VEGF-D, Flt-1, PIGF, βFGF); Vascular Injury Panel 2 V-Plex (SAA, VCAM-1, ICAM-1); Human Chemokine Panel 1 (MCP-1, MCP-4, Eotaxin, IP-10, MDC, Eotaxin-3, TARC, MIP-1α, MIP1β, IL-8). Samples were diluted and loaded into each well before reading by a SECTOR Imager 6000 Plate Reader (MSD Mesoscale Discovery, USA) according to the manufacturer’s instructions. Data was analyzed using MSD Discovery Workbench software.

### Histopathology

Before histological analysis, brains of mice (untreated controls, DEM, aREM, fREM) were postfixed in 4% PFA overnight and embedded in paraffin. Then, 8 μm coronal sections were obtained at the levels of 161, 181, 209 and 221. Demyelination was evaluated using Luxol fast blue staining with cresyl violet, and axonal pathology was examined by Bielschowsky staining. Immunocytochemistry was performed on paraffin sections as described before [16] without antigen retrieval using antibodies against apolipoprotein C-II (MyBioSource, MBS2006755), TIMP-1 (R&D systems, AF980-SP), tyrosine protein kinase receptor UFO (Santa Cruz Biotechnology, sc-166269), Mac3 (Becton & Dickinson #553322), NG2 (Millipore AB 5320), and CNP (Sternberger Monoclonals SMI 91).

### Proteomics and parallel reaction monitoring (PRM)

#### Sample preparation and qualitative mass spectrometry (proteomics-discovery step)

20 uL of 10 CSF samples for each of the groups: controls, RR, SP and PPMS were pooled separately. Proteins were ethanol/acetone precipitated, redissolved in 7M, 2M thiourea, 20mM DTT. Protein amount was estimated using Qubit Protein Assay (Thermo Fisher Scientific). Following alkylation with IAA proteins were digested with trypsin (50:1 ratio) overnight at 37°C. Peptides were RP purified using home-made columns of R2 and R3 (Applied BiosystemsTM). Purified peptides were redissolved in 0.1% formic acid. One μg of peptide sample in 5 μL was loaded on a precolumn (3 cm long, 100 μm ID, packed with 5 μm C18 RP material (Dr. Maisch, Ammerbuch-Entringen, Germany) using nano-Easy LC (Thermo Fisher Scientific). Peptides were separated on a 18 cm long, 75 μm ID analytical column packed with 3 μm C18 material (Dr. Maisch, Ammerbuch-Entringen, Germany) using 100 min gradient of 90% ACN/0.1% FA (solvent B): 0% to 10% in 5 min, 10% to 34% (linear gradient) in 77 min, 34% to 100% in 10 min and 8 min at 100%. Peptides were fragmented and detected in Q-Exactive mass spectrometer (Thermo Fisher Scientific, Bremen, Germany). The MS settings: Full MS: Resolution at 60000, AGC target 3e6, Maximum IT 50 ms. MS2: ten MSMS events at 15000 resolution, AGC target 3e6, Maximum IT 50 ms, isolation window 1.0 m/z, NCE: 28. Raw data were processed and proteins identified using ProteomeDiscoverer software v1.4 (Thermo Fisher Scientific).

#### Selection of peptides for PRM

PeptideAtlas repository (http://www.peptideatlas.org), available in-house data and data generated during present work (see above) were used to select number of peptides for each of the 98 proteins. The main criteria for the selection were the high frequency of observation in CSF samples and a length of peptide (up to 20 AA). Subsequently, aided by Skyline software (https://skyline.ms; v. 3.5.0.9319; MacCoss Lab, Department of Genome Sciences, UW) a list of precursor ions m/z (charge state 2 to 4+ per peptide, if relevant) for these peptides were generated and used to re-search for their presence in CSF pools (see above). Finally, the list was shortened to comprise up to 4 peptides per protein based on the intensity of detected peptides, and if not detected, then on the frequency of detection in the PeptideAtlas repository. Synthetic standard (SIS; crude preparation), heavy K and R labelled peptides, were purchased from JPT Peptide Technologies (www.jpt.com). Their quality was tested by mass spectrometry analysis.

#### Sample preparation for mass spectrometry/PRM

Proteins in 100 uL of individual CSF samples were ethanol/acetone precipitated and redissolved in 50 uL 3M urea/thiourea (3.5:1), 20 mM DTT (**S1 Table**). Protein amount was estimated by amino acid composition analysis. 10 μg (and if not available, less was taken and appropriate correction was later done to the final PRM results) was brought to final 140 uL, samples comprised in total 7M urea and thiourea (ratio 3.5 to 1) and 20 mM DTT. Cysteines were alkylated with 45 mM IAA and proteins digested with trypsin (50:1). After digestion, the same amount of SIS peptide mix was added to each sample and peptides were RP purified (as above). Purified peptides were dissolved in 0.1% FA (Solvent A).

#### PRM analysis

0.5 μg of peptide sample in 5 μL was loaded on a precolumn (3 cm long, 100 μm ID, packed with 5 μm C18 RP material (Dr. Maisch, Ammerbuch-Entringen, Germany) using nano-Easy LC (Thermo Fisher Scientific). Peptides were separated on a 18 cm long, 75 μm ID analytical column packed with 3 μm C18 material (Dr. Maisch, Ammerbuch-Entringen, Germany) using 62 min gradient of 90% ACN/0.1% FA (solvent B): 8% for 5 min, 8% to 30% (linear gradient) in 44 min, 30% to 100% in 5 min and 8 min at 100%. Peptides were fragmented and detected in Q-Exactive mass spectrometer (Thermo Fisher Scientific, Bremen, Germany). The MS settings: Full MS: Resolution at 120000, AGC target 3e6, Maximum IT 30 ms, scan range: 325–1200 m/z. PRM (10 reaction between Full MS scan): Resolution at 15000, AGC target 3e6, Maximum IT 15 ms, isolation window 1.1 m/z, NCE: 32. MS data were processed using Skyline. The low intensity of most of the detected peptides, originating from the investigated proteins resulted in poor MS2 spectra, therefore for the consistency, peptide relative quantification was performed using MS1 intensity of the precursor ion normalized to intensity of the corresponding SIS standard.

### Statistical analysis

Raw RNA expression data obtained by the microarray were normalized to the 75th percentile signal intensity and entities showing present call in all samples of a condition were kept. Differentially expressed genes were selected when passing the signal intensity filter (entities where at least 100 percent of samples in any 1 out of 4 conditions have values within cut-off) and showing at least 2-fold statistically significant change (ANOVA and Tukey HSD post-hoc test, with Benjamini-Hochberg Multiple Testing Correction p-value<0.05) between any of the groups. To search for known pathways containing a significant overlap with the relevant proteins, over-representation analysis from the Consensus Pathway Database (CPDB, http://cpdb.molgen.mpg.de/, Reslease 31), was performed. Ingenuity Pathway Analysis (IPA, http://www.ingenuity.com) software was used to identify upstream transcriptional regulators that can explain the observed transcription profile. Gene expression data was examined by the Upstream Regulator module of IPA. The prediction of potential regulatory networks was based on Ingenuity Knowledge Base, a very abundant collection of functional gene interactions obtained by manual curation of biomedical literature and biological databases.

If not otherwise stated, statistical tests were performed using Prism 5 software (GraphPath, USA, CA). MesoScale and peptide data were analyzed by ANOVA. All statistical tests were followed by an appropriate post hoc test and quantitative data are presented as mean ± SEM, and p<0.05 is considered significant. Pearson correlation was used to examine correlation between CSF proteins and biomarkers. All p-values were corrected for multiple-testing, using Benjamini-Hochberg.

## Results

### Differentially expressed genes in the de- and remyelinated corpus callosum

**Fig 2:**
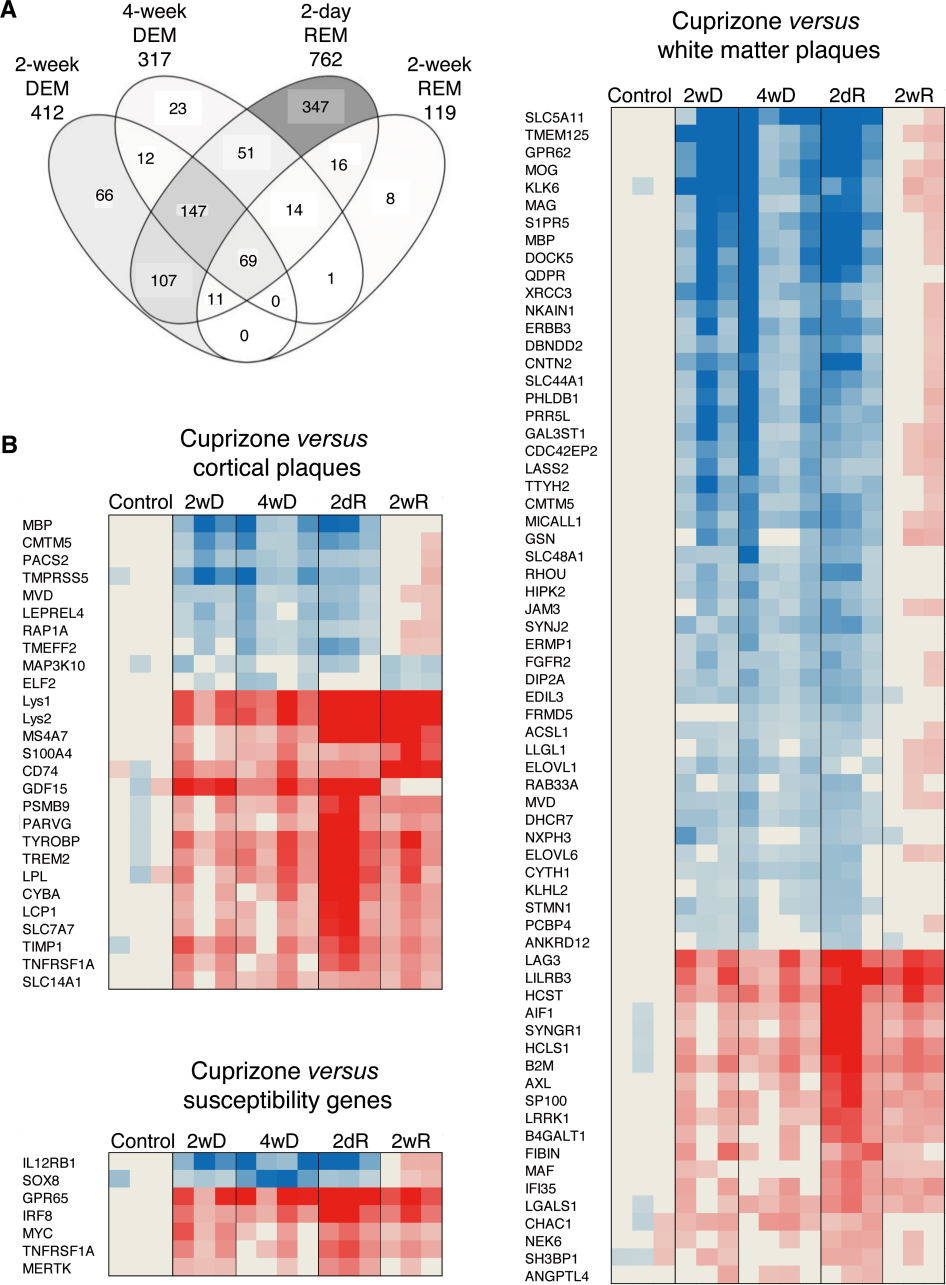
Gene expression during experimental de- and remyelination and orthologous genes in MS lesions. **(A)** Overlaps of differentially expressed (at least 2-fold change and statistically significant difference at corrected p<0.05) genes in four CPZ-treated experimental conditions compared to the untreated controls are visualized by Venn diagram. Intensity of filling grey colour indicates the relative percent of all genes shown. The transcriptome of demyelination was determined after 2 and 4 weeks of CPZ administration (2-week DEM, 4-week DEM); remyelination transcriptomes were determined 2 days and 14 days after suspending CPZ (acute and full remyelination; 2-day REM, 2-week REM). **(B)** Orthologous genes in MS and CPZ lesions were determined by comparing differentially expressed genes in white matter and cortical lesions of MS patients and MS susceptibility genes to transcriptome signatures of CPZ-induced demyelination at 2 and 4 weeks (2wD, 4wD), acute remyelination 2 days after suspending CPZ (2dR), and during full remyelination 2 weeks after suspending CPZ (2wR). The heatmaps represent the expression of orthologous: overlapping MS genes during demyelination and remyelination phases of the CPZ model.

By using stereomicroscope, the corpus callosum was cut out from brain sections 2 weeks and 4 weeks after feeding CPZ to mice (early and full demyelination), as well as 2 days and 2 weeks after suspending CPZ (acute and full remyelination) (**Fig 1**). The Agilent microarray identified 1239 differentially expressed genes in the de- and remyelinated corpus callosum (**Fig 2A**). The highest number of differentially expressed genes was observed during acute remyelination (n = 762), followed by early demyelination (n = 412) and full demyelination (n = 317); the number of differentially expressed genes was markedly reduced by the time of full remyelination (n = 119) (**Fig 2A**). The majority of differentially expressed genes during 2 and 4 weeks demyelination overlapped (n = 228); only 28% of differentially expressed genes during late, the 4-week demyelination were different from genes of 2-week demyelination (**Fig 2A**). Acute remyelination in the CPZ model was accompanied by a large number of newly activated genes: 63% of the differentially expressed genes were different from activated genes during 4-week demyelination. The majority of these genes were also different from late (2-week) remyelination: only 14% were shared with late remyelination. In contrast to different gene expression between demyelination and acute remyelination, only 7% of differentially expressed genes related to late remyelination were different from acute remyelination (**Fig 2A**).

### Orthologous genes of murine and MS lesion transcriptomes

**Fig 3:**
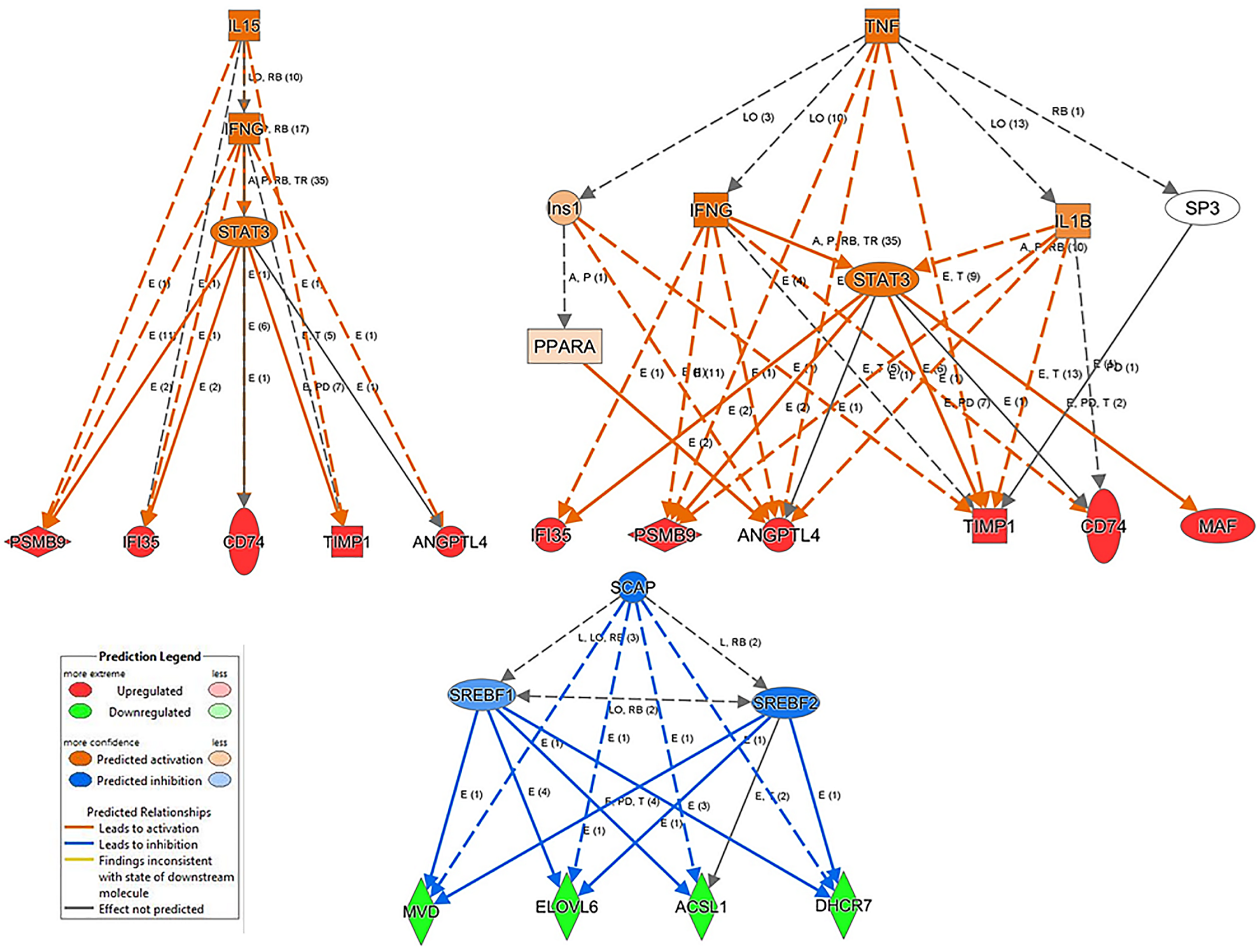
Upstream regulators of up- and down-regulated orthologous genes in the cuprizone model and MS lesions. Upstream regulators of up- and down-regulated orthologous genes were determined by Ingenuity Pathway Analysis. The experimentally observed gene expression tendencies are shown by the bottom line of nodes of the graphs (red increased, green decreased expression), while the networks above these genes displays the predicted regulatory networks might be responsible for the detected transcription effect. Orange color in the regulatory network indicates positive and blue color indicates negative regulatory effects. Marks on the graph edges shows the type of functional interactions (E: expression, LO: localization, A: activation, TR: translocation, T: transcription, RB: regulation of binding, PD: protein-DNA binding, P: phosphorylation) and the number of the supporting evidences in Ingenuity Knowledge Base.

Next, we linked differentially expressed genes in MS lesions to genes differentially expressed in experimental de- and remyelinated lesions (**Fig 2B**): we compared published datasets of active, chronic active, chronic, early and late lesions in the white matter, and cortical grey matter transcriptomes in the MS brain [17–20] to the 1239 differentially expressed genes in the CPZ model. We identified 91 overlapping orthologous genes: 26 of these were differentially expressed in cortical lesions, and 65 were differentially expressed in white matter lesions. Two of the genes (*Mbp* and *Cmtm5*) were differentially expressed in both kinds of lesions (**Fig 2B**). We also found 7 susceptibility genes identified by GWAS, which were differentially expressed in the de- and remyelinated corpus callosum (**Fig 2B**). Out of the overlapping orthologous WM and cortical MS lesion genes, 42 genes were related to experimental demyelination, and 41 genes were related to remyelination. Pathway analysis of the orthologous genes was performed. Out of 18 significantly regulated, demyelination-related pathways in MS lesions, 15 were related to lipid metabolism; in contrast, only 2 of the 10 remyelination-related pathways were related to lipid metabolism (**Table 1**). This differential representation of lipid metabolism pathways was also confirmed by the IPA (data not shown). Pathway analyses of the 98 orthologous genes suggested the importance of molecules TYROBP, TREM2, TNFRSF1A, CYBA, and PSMB9 besides molecules related to lipid metabolism (**Table 1**). When upstream regulators of the orthologous genes were analyzed, three pathways emerged: up-regulated IL-15/IFN-γ/STAT3 and TNF/IFN-γ/STAT3/IL-1β, and down-regulated SCAP/SREBF1/SREBF2 (**Fig 3**). The differentially expressed genes of the downregulated SCAP/SREBF1/SREBF2 orthologous pathway (*Mvd*, *Elovl6*, *Acsl1*, *Dhcr7*) were all downregulated during 2-weeks and 4-weeks demyelination, and acute remyelination. The differentially expressed genes of the upregulated IL-15/IFN-γ/STAT3 and TNF/IFN-γ/STAT3/IL-1β, orthologous pathways were all upregulated during demyelination, acute and full remyelination, but with different kinetics: three genes (*Timp1*, *Psmbp9*, *Ifi35*) peaked during acute remyelination, *Cd74* peaked during full remyelination, and *Angptl4* reached a plateau from 2-weeks demyelination till full remyelination.

**Table 1:** Pathways of orthologous genes differentially expressed in both experimental de/remyelination and MS lesions.

Pathway	p-value	q-value	source	members
***WM + cortical MS lesion versus experimental demyelination***				
stearate biosynthesis	0.0004	0.006	HumanCyc	ACSL1; ELOVL1
cholesterol biosynthesis	0.0006	0.006	Wikipathways	MVD; DHCR7
PPAR signaling pathway	0.0006	0.006	KEGG	LPL; ACSL1; ANGPTL4
synthesis of very long-chain fatty acyl-CoAs	0.0008	0.006	Reactome	ACSL1; ELOVL1
omega-3 fatty acid metabolism	0.0009	0.006	EHMN	ACSL1; ELOVL1
activation of gene expression by SREBP (SREBF)	0.001	0.006	Wikipathways	MVD; DHCR7
metabolism of lipids and lipoproteins	0.001	0.006	Reactome	SYNJ2; ACSL1; DHCR7; LPL; MVD; ELOVL1
fatty Acyl-CoA biosynthesis	0.001	0.006	Reactome	ACSL1; ELOVL1
cholesterol biosynthesis	0.002	0.0065	Reactome	MVD; DHCR7
activation of gene expression by SREBF (SREBP)	0.002	0.0065	Reactome	MVD; DHCR7
superpathway of cholesterol biosynthesis	0.002	0.007	HumanCyc	MVD; DHCR7
regulation of cholesterol biosynthesis by SREBP (SREBF)	0.002	0.007	Reactome	MVD; DHCR7
amb2 Integrin signaling	0.0025	0.007	PID	RAP1A; JAM3
omega-6 fatty acid metabolism	0.003	0.009	EHMN	ACSL1; ELOVL1
fatty acid beta oxidation	0.005	0.01	Wikipathways	ACSL1; LPL
triglyceride biosynthesis	0.006	0.01	Reactome	ACSL1; ELOVL1
class I PI3K signaling events	0.009	0.02	PID	CYTH1; RAP1A
integrated breast cancer pathway	0.009	0.02	Wikipathways	XRCC3; RAP1A
***WM + cortical MS lesion versus experimental remyelination***				
glial cell differentiation	0.0002	0.006	Wikipathways	MBP; MAG
osteoclast differentiation	0.0003	0.006	KEGG	TYROBP; TNFRSF1A; CYBA; TREM2
other semaphorin interactions	0.001	0.02	Reactome	TYROBP; TREM2
RANKL	0.002	0.02	NetPath	TYROBP; TREM2
basigin interactions	0.002	0.02	Reactome	SLC7A7; MAG
spinal cord injury	0.003	0.02	Wikipathways	MBP; AIF1; MAG
hiv-1 nef: negative effector of fas and tnf	0.008	0.04	BioCarta	GSN; TNFRSF1A
cardiac hypertrophic response	0.0085	0.04	Wikipathways	FGFR2; TNFRSF1A
DAP12 signaling	0.009	0.04	Reactome	TYROBP; FGFR2; TREM2
caspase cascade in apoptosis	0.009	0.04	PID	GSN; TNFRSF1A

### Identification of 19 proteins of the 98 orthologous genes in the full CFS proteome (cohort 1)

We then examined, if proteins reflecting the 98 orthologous genes could be detected in the proteome of human CSF obtained from patients with RRMS, SPMS and primary progressive MS (**Fig 1**). Twenty μl of CSF from 10 subjects in each of the 3 groups were pooled and examined by LC-MS/MS (discovery cohort). We detected 19 corresponding proteins out of the 98 orthologous differentially expressed genes in the full CSF proteome (**Table 2**).

**Table 2:** Orthologous cuprizone and MS lesion genes detected in the CSF by proteomics and selected for targeted proteomics.

Protein	Gene	Description	CPZ	MS	GWAS
***Detected by proteomics in the CSF***					
APOC2	APOC2	Apolipoprotein C-II	DEM up	up^1^	no
LEG1	LGALS1	Galectin-1	DEM up	up^2^	no
TIMP1	TIMP1	Metalloproteinase inhibitor 1	DEM up	up^1^	no
ATS1	ADAMTS1	Disintegrin and metalloproteinase	DEM down	up^2^	no
HEP2	SERPIND1	Heparin cofactor 2	DEM down	down^3^	no
MOG	MOG	Myelin-oligodendrocyte glycoprotein	DEM down	down^3^	no
CNTN2	CNTN2	Contactin-2	DEM down	down^3^	no
DBND2	DBNDD2	Dysbindin domain-containing protein 2	DEM down	up^2^ down^3^	no
UFO	AXL	Tyrosine-protein kinase receptor UFO	eREM up	up^2^	no
TREM2	TREM2	Triggering receptor expressed on myeloid cells 2	eREM up	up^1^	no
MAG	MAG	Myelin-associated glycoprotein	eREM down	up^2^	no
KLK6	KLK6	Kallikrein-6	eREM down	down^3^	no
DHPR	QDPR	Dihydropteridine reductase	eREM down	down^3^	no
FGFR2	FGFR2	Fibroblast growth factor receptor 2	eREM down	down^3^	no
GELS	GSN	Gelsolin	eREM down	down^3^	no
B2MG	B2M	b2-microglobulin	DEM, REM up	up^2^	no
LYSC	LYZ	Lysozyme C	DEM, REM up	down^2^	no
BGH3	TGFBI	Transforming growth factor,-beta-induced	-	up^2^	no
HPT	HP	Haptoglobin	-	up^2^	no
***Additional genes selected for targeted proteomics***					
ANGL4	ANGPTL4	Angiopoietin-related protein 4	DEM up	ac up	no
CD83	CD83	CD83 antigen	DEM up	-	no
GDF15	GDF15	Growth/differentiation factor 15	DEM up	ac, chr up	no
IGF1	IGF1	Insulin-like growth factor I	DEM up	-	no
S10A4	S100A4	Protein S100-A4	DEM up	-	no
SLAF5	CD84	SLAM family member 5	DEM up	-	no
CERS2	CERS2	Ceramide synthase 2	DEM down	-	no
CKLF5	CMTM5	CKLF-like MARVEL transmembrane domain-containing protein 5	DEM down	-	no
DHCR7	DHCR7	7-dehydrocholesterol reductase, pathway cholesterol biosynthesis	DEM down	-	no
EDIL3	EDIL3	EGF-like repeat and discoidin I-like domain-containing protein 3	DEM down	-	no
ELOV1	ELOVL1	Elongation of very long chain fatty acids protein 1	DEM down	-	no
ERBB3	ERBB3	Receptor tyrosine-protein kinase erbB-3	DEM down	-	no
IL12R1	IL12RB1	Interleukin-12 receptor subunit beta-1	DEM down	chr up	yes
IL33	IL33	Interleukin-33	DEM down	-	no
ITB4	ITGB4	Integrin beta-4	DEM down	-	no
LDLR	LDLR	Low-density lipoprotein receptor	DEM down	-	no
LPAR1	LPAR1	Lysophosphatidic acid receptor 1	DEM down	-	no
SYNJ2	SYNJ2	Synaptojanin-2	DEM down	-	no
AIF1	AIF1	Allograft inflammatory factor 1	eREM up	-	no
CNTF	CNTF	Ciliary neurotrophic factor	eREM up	-	no
HCST	HCST	Hematopoietic cell signal transducer	eREM up	-	no
IRF8	IRF8	Interferon regulatory factor 8	eREM up	-	yes
MYOF	MYOF	Myoferlin	eREM up	-	no
SNG1	SYNGR1	Synaptogyrin-1	eREM up	-	no
TNR1A	TNFRSF1A	Tumor necrosis factor receptor superfamily member 1A	eREM up	-	yes
TYOBP	TYROBP	TYRO protein tyrosine kinase-binding protein	eREM up	-	no
TEFF2	TMEFF2	Tomoregulin-2	eREM down	-	no
STMN4	STMN1	Stathmin	eREM down	-	no
MBP	MBP	Myelin basic protein	eREM down	-	no
G3ST1	GAL3ST1	Galactosylceramide sulfotransferase	eREM down	-	no
PSYR	GPR65	Psychosine receptor	DEM, REM up	ac, chr up	yes
UBD	UBD	Ubiquitin D	-	ac up	no

DEM: demyelination; REM: remyelination; eREM: early remyelination; chr: chronic; ac: acute;

^1^[19];

^2^[18];

^3^[17]

### Selection of 51 proteins for targeted proteomics and design of peptide library

Next, we designed a targeted proteomics approach (PRM) to quantify and compare the level of proteins of the 19 orthologous genes in additional CSF of relapsing and progressive MS (cohort 2). However, we considered that potential proteins might be missed in the CSF proteome because of the low volume of the examined CSF and low number of subjects in the LC-MS/MS analysis. Therefore, we selected 32 additional genes from the CPZ datasets for protein quantification in the CSF based on biological significance, cell surface expression, potential release upon cellular damage, and overlap with orthologous genes differentially expressed in cortical or WM lesions from patients with MS (**Table 2**). Out of the total 51 selected genes for targeted proteomics, 24 were expressed in WM lesions of MS brain, 9 genes were expressed in cortical lesions, and 4 were GWAS genes (**S2 Table**). For each of the 51 genes, 2–4 peptide sequences representing the corresponding proteins were designed based on in-house proteome data of human blood, CSF, brain and cell cultures (**S2 Table**).

### Measuring levels of the 129 peptides in 97 CSF samples (cohort 1 and 2)

By using the established peptide library representing the 51 proteins, we measured the levels of the 129 peptides in 97 individual CSF samples by PRM: 57 patients with progressive MS (30 PPMS, 27 SPMS) and 40 patients with RRMS. We could detect 24 peptides of 10 proteins in all 97 CSF samples (**S3 Table**).

### Differential regulation of 4 peptides representing 4 proteins in 97 CSF samples

**Fig 4:**
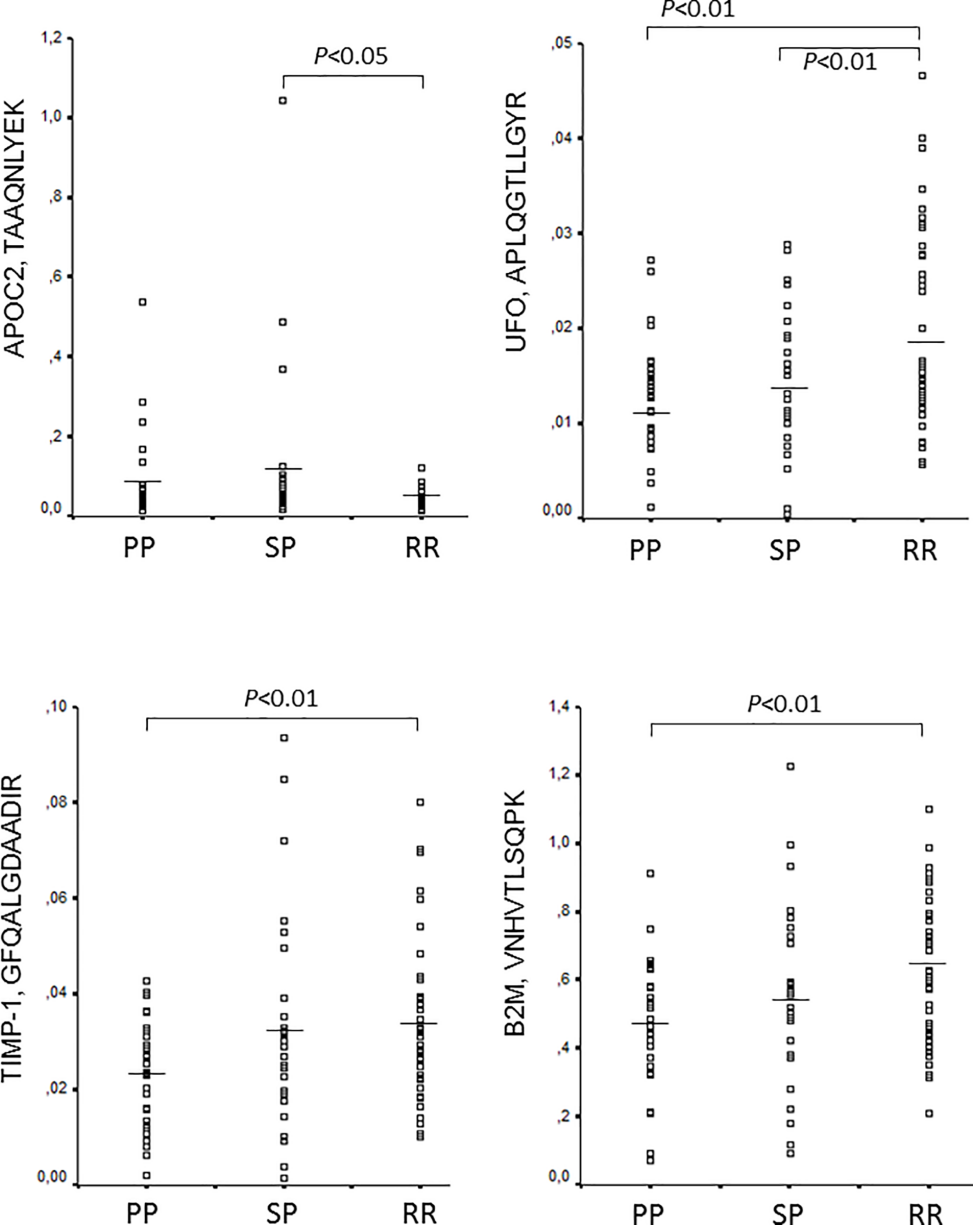
Differential regulation of 4 peptides in the CSF of patients with relapsing and progressive MS. Level of peptides relative to the respective stable isotope standard is indicated by scatter plots, and was quantified by targeted proteomics, parallel reaction monitoring in the CSF of patients with relapsing-remitting MS (RRMS, n = 40), secondary progressive MS (SPMS, n = 27), and primary progressive MS (PPMS, n = 30). (ANOVA, corrected for multiple-testing, using Benjamini-Hochberg). *See also S1 and S3 Tables*.

The levels of the detected 24 peptides normalized to synthetic standards (**S3 Table**) were then compared among the 3 MS subgroups. Four peptides representing 4 proteins were differentially regulated: tissue inhibitor of metalloproteinase-1 (TIMP-1, GFQALGDAADIR), apolipoprotein C2 (APOC2, TAAQNLYEK), tyrosine-protein kinase receptor UFO (UFO, APLQGTLLGYR), and beta2 microglobulin (B2M, VNHVTLSQPK) (**Fig 4, S4 Table**). All these proteins were detected in the CSF in the discovery phase by LC-MS/MS (**Table 2**). APOC2 peptide was upregulated in SPMS compared to RRMS. The other 3 peptides (TIMP-1, UFO, B2M) were downregulated in PPMS compared to RRMS. In addition, UFO peptide was also down-regulated in SPMS (**Fig 4**).

### Correlation of TIMP-1, APOC2, UFO, and B2M levels with inflammatory markers and neurofilament in the CSF

**Fig 5:**
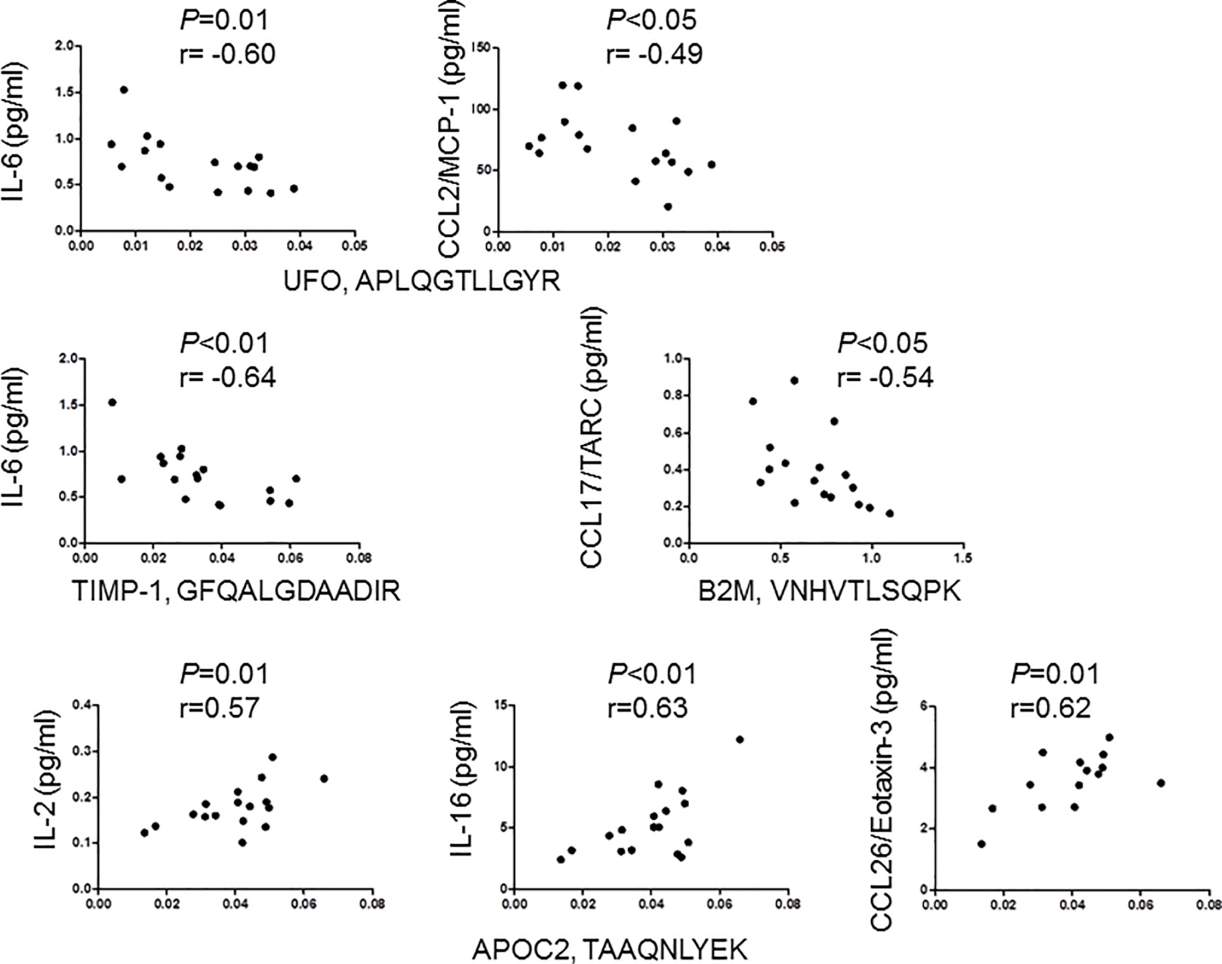
Correlation of peptide levels with pro-inflammatory markers in the CSF of patients with relapsing-remitting MS. The concentration of the indicated biomarkers was examined by MesoScale Discovery technique, and the levels of peptides were quantified by parallel reaction monitoring in the CSF of 17 patients with RRMS.

We next examined, if the level of the 4 peptides differentially expressed in MS subgroups correlate with concentration of 36 neuroinflammatory markers and neurofilament light chain (NF-L) in the CSF of 17 patients with RRMS. The level of UFO peptide APLQGTLLGYR and TIMP-1 peptide GFQALGDAADIR negatively correlated with the concentration of IL-6. Peptide APLQGTLLGYR of UFO also negatively correlated with concentration of CCL2/MCP-1. Peptide VNHVTLSQPK of B2M also negatively correlated with the concentration of CCL17/TARC. In contrast, peptide TAAQNLYEK of APOC2 showed positive correlation with the concentration of IL-16, IL-2 and CCL26/eotaxin-3 (**Fig 5**). NF-L showed no correlation with the 4 peptides.

We also examined the correlation among the 4 peptides in the CSF of the same 17 patients with RRMS. We found that B2M correlated with levels of both UFO (p<0.0001, r = 0.91) and TIMP-1 (p<0.01, r = 0.71), and we also found correlation between TIMP-1 and UFO (p<0.01, r = 0.64).

### Genes and proteins of TIMP-1, APOC2, UFO, and B2M are over-expressed during acute remyelination

**Fig 6:**
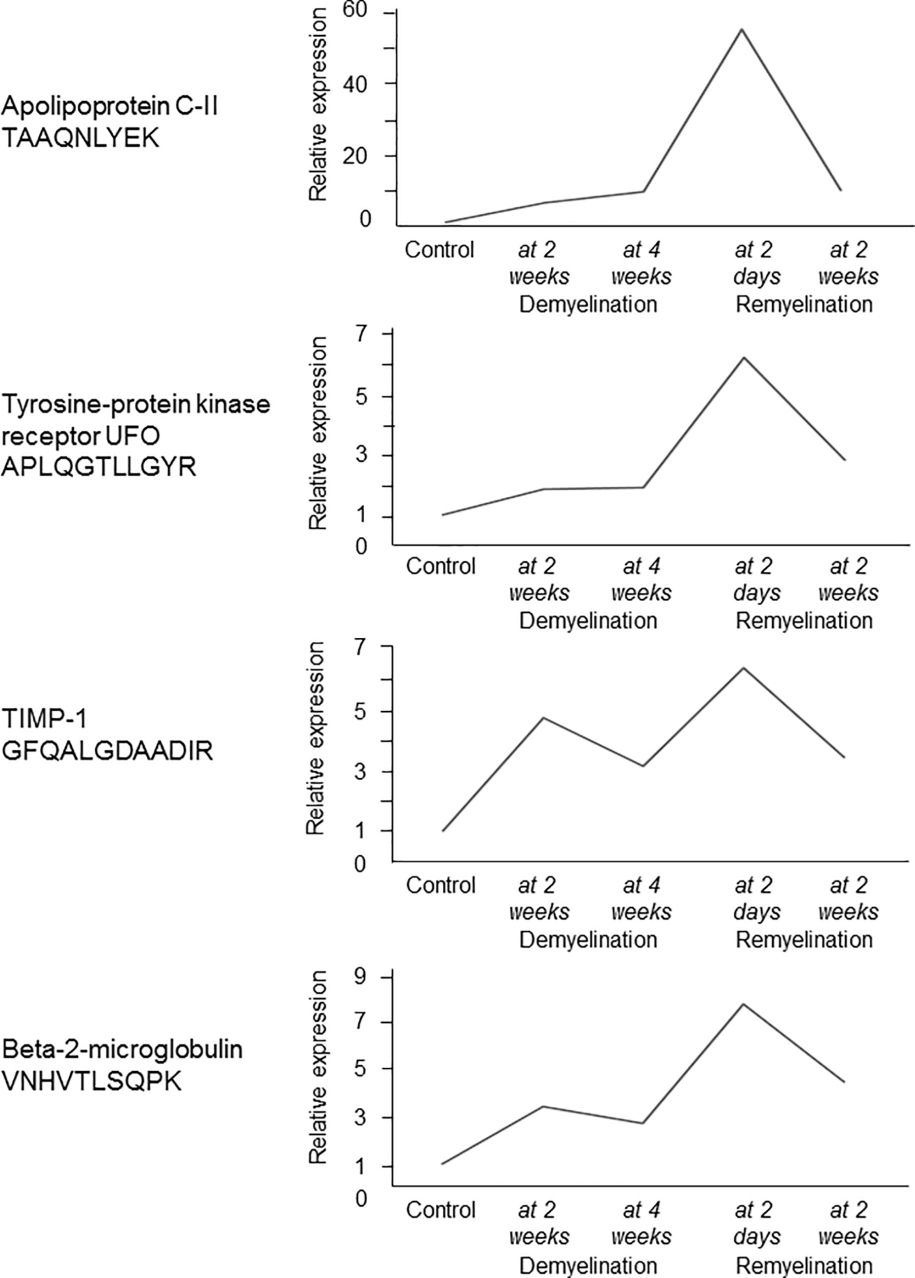
Experimental de- and remyelination kinetics of 4 genes with differentially regulated orthologues in the MS CSF. Relative expression of orthologous mouse genes of the 4 proteins was examined in the corpus callosum of control mice and during different phases of CPZ-induced demyelination (2 weeks, n = 3; and 4 weeks, n = 4), and acute and full remyelination (2 days after suspending CPZ, n = 3; and 14 days after suspending CPZ, n = 3) by 4x44K Agilent Whole Mouse Genom Expression Microarray. Mean of relative expression is shown at these time points.

To investigate if the expression of the 4 orthologues (TIMP-1, APOC2, UFO, and B2M) is related to de- or remyelination, and explore the dynamics of expression, we examined changes in the gene and protein expression of the 4 orthologues during experimental de- and remyelination in the CPZ model. All four genes peaked during acute remyelination, and the gene encoding TIMP1 was also upregulated during demyelination (**Fig 6**).

**Fig 7:**
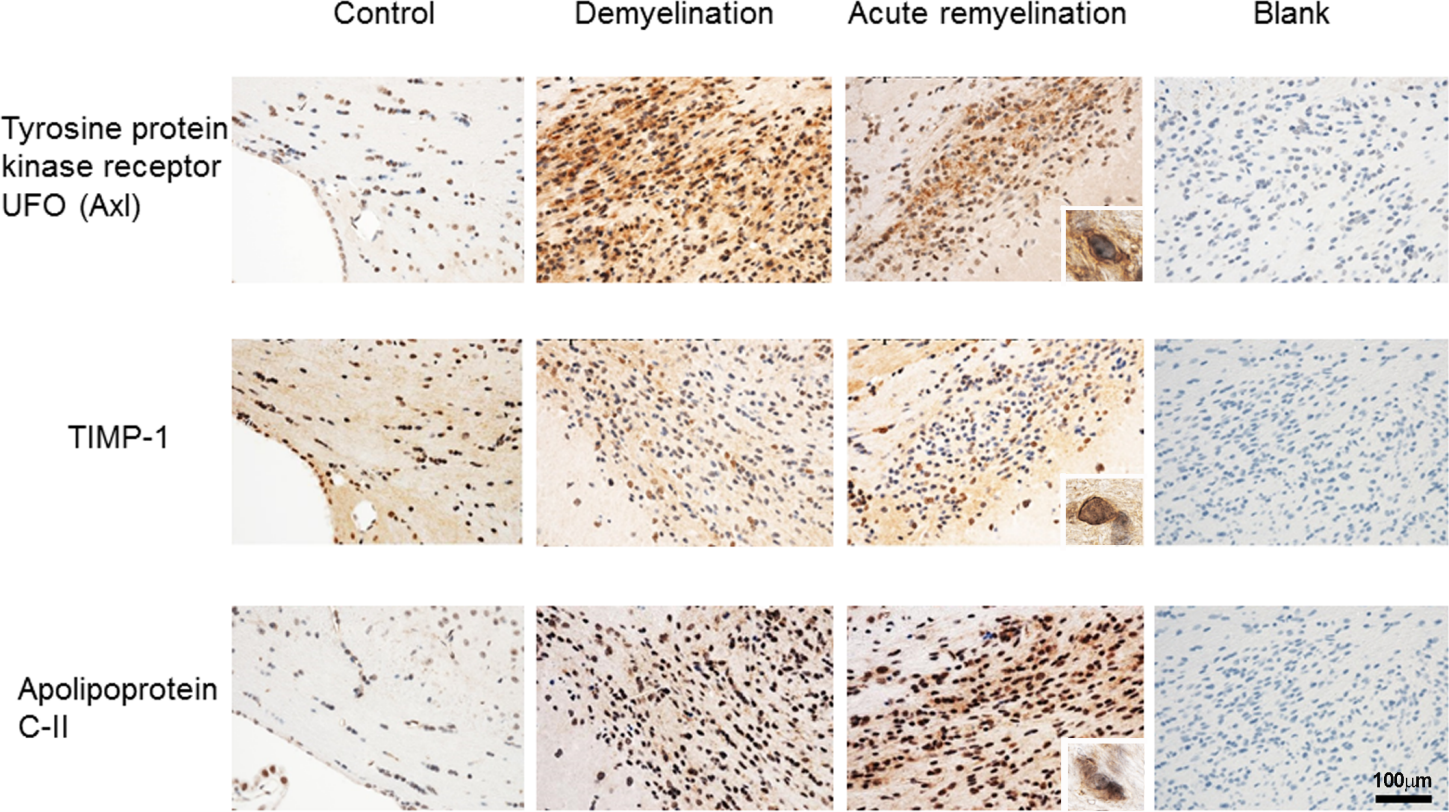
Expression of differentially regulated orthologous proteins in the CSF of patients with MS during CPZ-induced de- and remyelination. Protein expression of the indicated proteins have been examined by immunohistochemistry in the corpus callosum of controls, 4 weeks after CPZ administration (demyelination), and 2 days after suspending CPZ (acute remyelination). Blank indicates no antibody. Magnification bars represent 100 micrometer.

To examine the protein expression and validate microarray data, we examined the expression of TIMP-1, APOC2, and UFO by immunohistochemistry in different tissues and in the brain of mice during CPZ-induced de- and remyelination. In the normal mouse brain, we found some expression of UFO within large neurons and minor reactivity on glia (most likely astrocytes) in the pons and CC (**Fig 7**). In mice treated with CPZ, we found mildly increased immunoreactivity in neurons in the pons (data not shown). Within the demyelinating CC, we found clear reactivity on macrophages/microglia; similar reactivity was seen during acute remyelination (**Fig 7**). These data suggest that major up-regulation of UFO within the CPZ lesions is due to its expression in macrophages/microglia.

We found expression of TIMP-1 on the nuclear membrane of large neurons and minor reactivity in glia in the control animals (data not shown). In the CPZ lesions, we found reactivity in some cells, which by morphology are most likely oligodendrocytes or OPCs; this reactivity was increased during demyelination and acute remyelination in the CC, but not in the pons (**Fig 7**).

In the control brain, APOC2 immunoreactivity was seen in neurons and prominently in a subset of endothelial cells of vessels (data not shown). In the CPZ lesions, expression in endothelial cells was low or absent, but there was a variable degree of granular reactivity in macrophages, which was more pronounced during acute remyelination compared to demyelination (**Fig 7**).

## Discussion

Here, we applied a multi-omics approach to examine molecular pathways related to cellular responses that are associated with de- and remyelination in MS lesions, and used these findings to discover molecules that are differentially regulated in the CSF proteome among MS subtypes. We first examined the transcriptome signature of experimental de- and remyelination, and compared these to MS lesion transcriptomes in order to identify de- and remyelination related pathways and molecules in MS. We then examined the CSF proteome in a discovery MS cohort by LC-MS/MS, and protein products of the overlapping orthologous genes were screened in the CSF proteome. Based on these data, we designed 129 peptides that represented the orthologous genes, measured them in 97 CSF samples of additional MS patients (cohort 1 and 2), and compared their levels between relapsing and progressive MS subtypes. Peptides that were differentially regulated between RRMS and progressive MS were correlated with molecular markers representing inflammation, BBB dysfunction, and axonal damage. Finally, the expression kinetics of orthologous genes of the differentially regulated proteins were examined in the CPZ model, and related to experimental demyelination, acute and full remyelination.

In the animal model, the majority of differentially expressed genes during 2 and 4 weeks demyelination overlapped, and almost all differentially regulated pathways were related to lipid metabolism. We found the greatest rearrangement of gene expression during acute remyelination, indicating a rapid activation of genes. Many of the differentially regulated pathways/genes during acute remyelination, i.e. 2 days after suspending CPZ were associated with immune responses. Since adaptive immune responses are absent or minor in the CPZ model, this may reflect rapid microglia responses during acute remyelination. By the time of late remyelination (2 weeks), the majority of these genes (85%) were not differentially expressed, and only 7% were newly expressed.

We next compared the transcriptome signatures of experimental de- and remyelination to a wide variety of MS lesion transcriptomes: active, chronic active, chronic lesions, early and late lesions, cortical and white matter lesions [17–20]. The orthologous CPZ/MS genes represented pathways of lipid metabolism, cell death and survival, and inflammatory responses.

Besides lipid metabolism, pathway analysis of the orthologous CPZ/MS genes suggested the importance of TNFRSF1A encoding TNF receptor 1, CYBA (cytochrome b-245 alpha subunit) required for enzymes producing reactive oxygen species [21], PSMB, a proteasome subunit that is important in antigen presentation and regulation of apoptosis [22], and TREM2, TYROBP. In the CNS, TREM2 is expressed exclusively by microglia [23], and is crucial in basic microglia functions: ligation of TREM2 induces downstream signaling through its signaling partner TYROBP/DAP12 [24], and results in cellular activities in microglia including promotion of phagocytosis, survival, proliferation and inflammatory responses [25–27]. TREM2 through mTOR signaling is also important in maintaining the metabolic state of microglia and may serve as a sensor of stress [28]. Loss-of function mutations in TREM2/TYROBP/DAP12 cause myelin loss Nasu-Hakula disease indicating their role in maintaining CNS myelin integrity [25]. Thus, in both MS and in the cuprizone model, the upregulated TREM2/TYROBP pathway may indicate microglia activation reacting to myelin damage and/or apoptosis of oligodendrocytes. Indeed, in Trem2-deficient mice, activation of microglia and myelin clearance is deficient in the cuprizone model, and axonal injury is increased [29]. This may be explained by inability to sense myelin damage. In EAE, TREM2 is upregulated in microglia, and blocking TREM2 exacerbates disease [30]. These animal models may suggest a beneficial role of TREM2 in demyelinating diseases, and considering our data indicating TREM2/TYROBP as an orthologue CPZ/MS pathway, upregulation of TREM2/TYROBP may be also beneficial in MS. However, in recent neurodegenerative models with early pathology, targeting the TREM2/TYROBP-APOE pathway restored the homeostatic signature of microglia, and reduced neurodegeneration and neuroinflammation [27,31,32]. We also found that Apoc2 was upregulated in the de- and remyelinating corpus callosum in the CPZ model, and APOC2 was detected in the CSF of patients with MS. One of the mechanisms of sensing stress by TREM2 on microglia may be binding to phosphatidylserins and other phospholipids on the surface of stressed or apoptotic cells [25,33]. TREM2 is also able to bind apolipoproteins, i.e. APOE, APOA1, APOA2, APOD and APOE [25]. If APOC2 can be another binding ligand for TREM2 is unclear.

Upstream regulators of the orthologous CPZ/MS genes focused attention on several additional molecules: upregulated pathways of IL-15, IFN-γ, STAT3, TNF, and IL-1β; and down-regulated pathways of SCAP. IL-15 enhances the cytotoxic effect of lymphocytes; it is elevated in the serum and CSF of patients with MS and expressed on astrocytes in MS lesions [34,35]. SCAP (sterol regulatory element binding protein cleavage activation protein) control cholesterol synthesis [36]. In the upstream regulator orthologous pathways, all genes in the SCAP pathway were downregulated during demyelination (*Mvd*, *Elovl6*, *Acsl1*, *Dhcr7*); in contrast, in the upregulated inflammatory pathways three out of the five genes peaked during acute remyelination (*Timp1*, *Psmbp9*, *Ifi35*), one peaked during full remyelination (*Cd47*), and one gene reached a plateau during early demyelination that persisted during acute remyelination (*Angptl4*). These data indicate again that differentially expressed orthologous MS genes related to remyelination are associated with inflammatory responses most probably by microglia.

Next, we used the CPZ and the orthologous MS gene data sets for identifying molecules that are differentially expressed in the CSF of relapsing and progressive MS by using a multi-omics method. We applied a two-step approach of CSF proteomics: (i) we first identified protein products of orthologous genes in the CSF proteome, and (ii) used a targeted quantitative approach to validate and compare the levels of specific proteins among MS subtypes. Using this approach, we focused on those proteins that were differentially expressed in MS lesions as genes, and were related to de- and remyelination. We identified 4 proteins differentially regulated between relapsing and progressive MS: UFO, TIMP-1, APOC2, and B2M.

One of the overlapping CPZ/MS gene orthologues, UFO protein, a receptor tyrosine kinase belonging to the family of TAM receptors, was upregulated in the CSF proteome of patients with relapsing compared to progressive MS. In the CPZ model, UFO protein was upregulated on macrophages/microglia in the demyelinating and acute remyelinating corpus callosum, while expressed at low levels in the normal mouse brain. This pattern of upregulation and cellular source is compatible with the function of UFO, as its plays a role in clearing myelin debris and apoptotic cells [37]. In the absence of Axl and/or its ligand growth-arrest-specific protein 6 (Gas6), remyelination is delayed, axonal damage is more severe, and inflammation is more pronounced [38–41]. We also found that higher level of UFO was associated with lower CCL2/MCP-1 concentrations in the CSF. CCL2/MCP-1 regulates the migration of monocytes/macrophages, and both are upregulated in MS lesions [42,43]. Higher level of UFO in the CSF of RRMS was also associated with lower concentration of IL-6 that disrupts the integrity of the BBB, and promotes autoimmune CNS response [44]. UFO-deficient (Axl-KO) mice had also more severe clinical course of EAE, which indicates a protective role of UFO in the inflammatory model of MS [37]. Altogether, these data support the notion that UFO may contribute to tissue repair and less inflammation both in MS and experimental demyelination partially by promoting phagocytosis by microglia and reducing inflammation.

Another overlapping CPZ/MS gene orthologue, TIMP-1 was also upregulated in the CSF proteome of relapsing versus primary progressive MS. In the CPZ lesions, we found reactivity in cells, which by morphology are most likely oligodendrocytes or OPCs; this reactivity was increased during demyelination and further increased during acute remyelination. Besides regulating MMP-9 [45,46], TIMP-1 also exhibits a trophic, growth factor function: it promotes astrocyte recovery [47], OPC differentiation [48], and activates PI3K and Akt signaling [49]. TIMP-1 is also expressed in immature astrocytes [48]. We may speculate that our observation of TIMP-1 expression on oligodendrocytes/OPCs may be related to their differentiation and survival during demyelination and especially during remyelination. In line with our results, *Timp-1* mRNA has been described to be upregulated during demyelination and in the acute remyelination phase [50]. TIMP-1 is upregulated in cortical MS lesions [18], and its restricted expression promotes EAE pathology [51]. Low levels of TIMP-1 and high MMP-9 concentration in both CSF and serum may be a marker of MRI disease activity, and their ratio may estimate the integrity of the BBB [52,53]. We also found that levels of TIMP-1 in the CSF of RRMS negatively correlated with the concentration of IL-6, which may support the beneficial role of elevated TIMP-1 in controlling inflammation and possibly BBB damage, similar to UFO.

A third orthologue, B2M was also upregulated in relapsing versus primary progressive MS. B2M constitutes the light chain of MHC I proteins that are expressed by all nucleated cells. It is released during metabolism and degradation, and thereby reflects cell membrane removal and cellular turnover. The presence of B2M in the CSF may be a CNS inflammatory or tumor marker [54]. In our study, the level of B2M showed a week negative correlation with CSF concentration of CCL17/TARC that induces chemotaxis of T cells through CCR4. In a recent study, we found higher concentration of CCL17/TARC in SLE-associated NMOSD, and CCL17/TARC correlated with AQP4-antibody, anti-nucleosome, anti-dsDNA, and CXCL10/IP-10 levels [55]. Increased B2M gene expression in CPZ lesions may also indicate microglia responses related to demyelination and acute remyelination; such responses may be beneficiary by removing myelin debris and limiting inflammation.

In summary, the levels of UFO, TIMP-1 and B2M were higher in RRMS compared to progressive MS types; they correlated negatively with pro-inflammatory markers in the CSF; and also showed a strong positive correlation with each other.

In contrast to these three orthologues, the fourth orthologue, APOC2 was upregulated in the CSF proteome of progressive compared to relapsing MS, and this molecule is directly related to lipid metabolism in the brain [56]. APOC2 is a cofactor for the activation of lipoprotein lipase that mediates hydrolysis of triglycerides, and plays an important role in the transport of lipids in the CNS [57,58]. Apolipoprotein C2 deficiency is related to a lipid encephalopathy and spinocerebellar ataxia type 12 [59,60]. In contrast to the other three orthologues, APOC2 level in the CSF showed a strong positive correlation with the concentration of proinflammatory IL-2, IL-16, and CCL26/eotaxin-3. In MS and EAE, IL-16 is an important chemotactic factor for CD4^+^ T cells, and has been observed within the perivascular infiltrates [61]. APOC2 has been identified as a potential risk gene for MS, although this finding has been questioned by others [62–64].

UFO, TIMP-1, B2M and APOC2 all had similar expression profiles in the CPZ model: the peak gene expression was observed during acute remyelination, and up-regulated proteins were found during demyelination and acute remyelination. Nevertheless, the cellular source of these molecules may be different: UFO and APOC2 showed reactivity with macrophages/microglia, while TIMP-1 was upregulated most likely on oligodendrocytes or OPCs. These data may indicate that although these proteins have been related to de- and remyelination, they reflect different cellular responses including microglia activation. Indeed, microglia-related 24 genes (surface markers and phagocytosis-related genes) peaked during acute remyelination in our study; nevertheless, the oligodendrocyte precursor cell gene of NG2 was also upregulated during acute remyelination (data not shown).

Finally, we used the CSF-PR 2.0 database to examine if these 4 proteins have been previously detected and quantitatively changed in the CSF proteome of patients with MS [65]. This database search indicated that all of these 4 proteins have been previously detected in the CSF proteome of patients with RRMS. The level of TIMP-1 was increased in the CSF proteome of patients with RRMS compared to controls with other neurological diseases and healthy subjects [66–68], but there was no difference comparing patients with early MS to clinically definite MS [69]. The level of UFO was not different in the CSF proteome of early MS or RRMS compared to other neurological disease controls [66,67,69]. The level of B2M was increased in the CSF of proteome of patients with RRMS compared to other neurological disease controls [66,67], while other studies did not find such differences [66,67]. The level of APOC2 was not different in the CSF proteome of patients with RRMS and other neurological disease controls [66,67].

## Conclusion

Our study used a comprehensive, translational multi-omics approach. The differential gene expression during experimental de- and remyelination was compared to MS lesion transcriptomes, and overlapping orthologues were screened at protein levels in the CSF proteome of MS patients. Finally, 129 peptides of selected 52 protein orthologues were quantitatively measured in 97 individual CSF, respectively. Their levels were compared between relapsing and progressive MS, were correlated with inflammatory markers in the CSF, and were related to gene and protein expression kinetics in experimental de- and remyelination. We could conclude that (i) experimental demyelination was mainly associated with altered lipid metabolism, while acute remyelination was associated with a profound change in gene activation. Many of the upregulated genes during acute remyelination were related to immune responses, and overlapped with orthologues in MS lesions. In fact, (ii) three out of four protein orthologues that were differentially regulated in the CSF proteome of relapsing and progressive MS were not directly related to myelin metabolism, but to pathways important in cell survival and immune responses. (iii) Since adaptive immune responses are absent or minor in the CPZ model, our data may indicate the role of microglia during acute remyelination, and different cellular responses promoting oligodendrocyte survival; the TREM2/TYROBP orthologous pathway may be important in such processes. (iv) These events were reflected in the CSF proteome of patients with MS. (v) Interestingly, the three CPZ/MS orthologues that were upregulated in the CSF proteome of relapsing compared to progressive MS, were all negatively correlated with inflammatory molecules, while positively correlated with each other. Although their cellular source maybe different, previous data indicated their protective role in experimental models: UFO (*Axl*) promotes remyelination and oligodendrocyte survival and limits inflammation [29–33,41]; TIMP-1 limits inflammation, blood-brain barrier damage, and promotes OPC differentiation [46–50]; B2M may indicate microglia activation related to removal of myelin debris and limiting inflammation in the cuprizone lesions; soluble B2M in the MS CSF may indicate increased cellular metabolism and degradation in the CNS. Thus, (vi) increased levels of UFO, TIMP-1 and B2M in the CSF of patients with relapsing MS may indicate different cellular processes in the brain related to de- and remyelination that aim reducing inflammation and promoting survival of oligodendrocytes and their precursor cells. (vii) These processes are either less pronounced in the brain, or less reflected in the CSF in progressive MS. (viii) The only orthologue that were upregulated in the CSF proteome of progressive versus relapsing MS was APOC2 that is related to cholesterol metabolism in the brain. In contrast to the other three orthologues, APOC2 level showed a strong correlation with the concentration of proinflammatory molecules IL-2, IL-16, and CCL26/eotaxin-3. This may indicate that myelin and lipid-related pathological events in the CNS may be more pronounced (or more reflected in the CSF) in progressive MS, nevertheless, they may be associated with inflammatory responses even in this phase.

This study is not without limitations. The CSF full proteome was examined in 30 patients, and only a low volume of samples were applied from each patient. However, we did not restrict the design of the peptide library only to these findings, and the targeted proteomics was done with a large number of individual CSF samples using higher volumes of CSF. Although we validated the protein expression of UFO, APOC2 and TIMP-1 by immunohistochemistry, the cell-specific expression was not addressed by specific antibodies. Nevertheless, this was not a major aim of the study. The role of these identified four molecules as potential CSF biomarkers should be validated in independent larger studies. Their biomarker potential and role in MS pathogenesis are reflected by previous studies indicating their presence in the CSF proteome of patients with RRMS, and upregulation of TIMP-1 and B2M in the CSF proteome of RRMS compared to controls [65–69].

## Supporting information

S1 Table Protein concentration (μg/mL) in the CSF of patients with primary progressive (PP), secondary progressive (SP) and relapsing-remitting (RR) multiple sclerosis. (PDF)

S2 Table Differentially expressed genes in the CPZ model selected for targeted proteomics in MS-CSF and their expression in MS lesions. (PDF)

S3 Table Uniformly detected proteins in 97 CSF samples of MS by targeted quantitative proteomics. (PDF)

S4 Table Level of peptides relative to the respective stable isotope standard in the CSF of patients with primary progressive (PP), secondary progressive (SP) and relapsing-remitting (RR) multiple sclerosis. (PDF)
