# Supplementary material for: Orthologous proteins of experimental de- and remyelination are differentially regulated in the CSF proteome of multiple sclerosis subtypes
Source: PLoS One. 2018 Aug 16;13(8):e0202530. doi: 10.1371/journal.pone.0202530 (PMC6095600; doi:10.1371/journal.pone.0202530)
Supplement: S1 Table — (PDF) [file pone.0202530.s001.pdf]

S1 Table

Protein concentration (µg/mL) in the CSF of patients with primary progressive (PP), secondary progressive (SP) and relapsing-remitting (RR) multiple sclerosis

PP	SP	RR
110	240	400
226,7	196,7	146,7
244,4	163,3	154,2
411,4	150	241,7
380	183,3	170,8
340	263,3	116,7
213,3	243,3	187,5
233,3	200	520,8
200	640	173,3
180	520	230
286,7	200	266,7
140	326,7	243,3
250	253,3	356,7
372	176,7	170
179,2	244,4	326,7
258,3	225	203,3
204,2	100	233,3
744	86,67	155,6
300	236,7	370
153,3	80	250
253,3	173,3	336,7
520,8	156,7	323,3
286,7	244,4	280
280	1190	300
196,7	180	350
143,3	136,7	226,7
196,7	446,4	276,7
226,7		246,7
250		256,7

380	156,7
	216,7
	176,7
	176,7
	153,3
	313,3
	110
	156,7
	196,7
	313,3
	356,7
