# Supplementary material for: Orthologous proteins of experimental de- and remyelination are differentially regulated in the CSF proteome of multiple sclerosis subtypes
Source: PLoS One. 2018 Aug 16;13(8):e0202530. doi: 10.1371/journal.pone.0202530 (PMC6095600; doi:10.1371/journal.pone.0202530)
Supplement: S2 Table — (PDF) [file pone.0202530.s002.pdf]

S2 Table

Differentially expressed genes in the CPZ model selected for targeted proteomics in MS-CSF and their expression in MS lesions

Gene	Protein	WM	GM	GWAS	Peptide sequences
ADAMTS1	A disintegrin and metalloproteinase with thrombospondin motifs 1				LGRPSEEDEELVVPELER<br>QGDVGGTCGVVDDEPRPTGK
AIF1	Allograft inflammatory factor 1	x			YSSDEDLPSKLEGFK<br>LIGEVSSGSGETFSYPDFLR
ANGPTL4	Angiopoietin-related protein 4	x			QSGLFEIQPQGSPPFLVNCK<br>MTSDGGWTVIQR
APOC2	Apolipoprotein C-II				ESLSSYWESAK<br>TAAQNLYEK<br>TYLPAVDEK
AXL	Tyrosine-protein kinase receptor UFO	x			CQLQVQGEPPEVHWLR<br>TATITVLPQQPR<br>APLQGTLLGYR
B2M	b2-microglobulin	x			VEHSDLSFSK<br>DWSFYLLYYTEFTPTEK<br>VNHVTLSQPK
CD83	CD83 antigen				GQNGSFDAPNERPYSLK<br>LQSIFPDFSK
CD84	SLAM family member 5				IHALGPNYNLVISDLR

					ADINTQADPYTTTK
CERS2	Ceramide synthase 2				APPNATLEHFYLTSGK<br>SRPLANGHPILNNNHR
CMTM5	CKLF-like MARVEL transmembrane domain-containing protein 5	x			DRHPEEGVVAELQGFAVDK<br>HPEEGVVAELQGFAVDK
CNTF	Ciliary neurotrophic factor				SDLTALTESYVK<br>NEADGMPINVGDGGLFEK
CNTN2	Contactin-2	x			FAQLNLAAEDTR<br>VISDTEADIGSNLR<br>LSLEDSGMYQCVAENK
DBNDD2	Dysbindin domain-containing protein 2	x			LPPLPR
DHCR7	7-dehydrocholesterol reductase, pathway cholesterol biosynthesis	x			LLVSGFWGVAR<br>YTAAVPYR
EDIL3	EGF-like repeat and discoidin I-like domain-containing protein 3	x			VTGVITQGAK<br>GNIDNNTPYANSFTPPIK
ELOVL1	Elongation of very long chain fatty acids protein 1	x			MEAVVNLYQEVMK<br>ALQQNGAPGIAK
ERBB3	Receptor tyrosine-protein kinase erbB-3	x			LTFQLEPNPHTK<br>GFSLLIMK
FGFR2	Fibroblast growth factor receptor 2	x			DAAVISWTK<br>TVLIGEYLQIK

					DSGLYACTASR
GAL3ST1	Galactosylceramide sulfotransferase	x			TASSTLLNILFR<br>ATAWNMLDSHLYR
GDF15	Growth/differentiation factor 15		x		ASLEDLGWADWVLSPR<br>EVQVTMCIGACPSQFR<br>TDTGVSLQTYDDLLAK
GPR65	Psychosine receptor			x	YLAVVYPLK<br>KVYQAVR
GSN	Gelsolin	x			EVQGFESATFLGYFK<br>QTQVSVLPEGGETPLFK<br>AVEVLPK<br>DSQEEEKTEALTSAK
HCST	Hematopoietic cell signal transducer	x			SPAQEDGK<br>VYINMPGR
HP	Haptoglobin				LRTEGDGVYTLNNEK<br>DIAPTLTLYVGK<br>YVMLPVADQDQCIR<br>VTSIQDWVQK
IL12RB1	Interleukin-12 receptor subunit beta-1			x	SPEVTLQLYNSVK<br>MEWETPDNQVGAEVQFR<br>FSVEQLGQDGR

IGF1	Insulin-like growth factor I				APQTGIVDECCFR<br>GFYFNKPTGYGSSSR<br>RLEMYCAPLKPAK
IL33	Interleukin-33				DFWLHANNK<br>TDPGVFIGVK<br>VDSSENLCTENILFK
IRF8	Interferon regulatory factor 8			x	SPDFEEVTDR<br>IVPEEEQK<br>DEVVQVFDTSQFFR
ITGB4	Integrin beta-4				VDKDCAYCTDEMFR<br>ALEHVDGTHVCQLPEDQK<br>LVFSALGPTSLR
KLK6	Kallikrein-6	x			KPNLQVFLGK<br>ESSQEQSSVVR<br>AVIHPDYDAASHDQDIMLLR<br>LSELIQPLPLER
LDLR	Low-density lipoprotein receptor				NVVALDTEVASNR<br>VFWTDIINEAIFSANR
LGALS1	Galectin-1	x			SFVLNLGK<br>FNAHGDANTIVCNSK<br>LNLEAINYMAADGDFK

LPAR1	Lysophosphatidic acid receptor 1				LTVSTWLLR<br>SENPTGPTEGSDR
LYZ	Lysozyme C		x		WESGYNTR<br>ATNYNAGDR<br>STDYGIFQINSR
MAG	Myelin-associated glycoprotein	x			LLGDLGLR<br>DTVQCLCVVK<br>SLELPFQGAHR
MBP	Myelin basic protein	x	x		YLATASTMDHAR<br>DTGILDSIGR<br>TQDENPVVHFFK
MOG	Myelin-oligodendrocyte glycoprotein	x			ALVGDEVELPCR<br>FSDEGGFTCFFR<br>DHSYQEEAAMELK
MYOF	Myoferlin				IPAHQVLYSTSGENASGK<br>SLGPPGPPFNITPR
QDPR	Dihydropteridine reductase	x			QSIWTSTISSHLATK<br>EGGLLTLAGAK<br>GAVHQLCQSLAGK
S100A4	Protein S100-A4		x		ALDVMVSTFHK<br>ELPSFLGK

SERPIND1	Heparin cofactor II				GGETAQSADPQWEQLNNK<br>QFPILLDFK<br>TLEAQLTPR
SYNGR1	Synaptogyrin-1	x			AGGAFDPYTLVR<br>DNPLNEGTDAAR
STMN1	Stathmin	x			ASGQAFELILSPR<br>SKESVPEFPLSPPK<br>ESVPEFPLSPPK
SYNJ2	Synaptojanin-2	x			YDVGSAAYDTSDK<br>GGASEEALSAVAPR
TGFBI	Transforming growth factor-beta-induced protein ig-h3				GRQHGPNVCAVQK<br>STVISYECCPGYEK<br>DGTPPIDAHTR<br>YLYHGQTLETLGGK
TIMP1	Metalloproteinase inhibitor 1		x		GFQALGDAADIR<br>FVYTPAMESVCGYFHR<br>EPGLCTWQSLR
TMEFF2	Tomoregulin-2		x		IGDTVTCVCQFK<br>IEVMSLGR
TNFRSF1A	Tumor necrosis factor receptor superfamily member 1A		x	x	ECESGSFTASENHLR<br>QNTVCTCHAGFFLR

TREM2	Triggering receptor expressed on myeloid cells 2		x		VVSTHNLWLLSFLR<br>VLVEVLADPLDHR
TYROBP	TYRO protein tyrosine kinase-binding protein		x		ITETESPYQELQGQR<br>SDVYSDLNTQRPYYK
UBD	Ubiquitin D				SEEWDLMTFDANPYDSVK<br>TKVPVQDQVLLLGSK
