# Supplementary material for: Orthologous proteins of experimental de- and remyelination are differentially regulated in the CSF proteome of multiple sclerosis subtypes
Source: PLoS One. 2018 Aug 16;13(8):e0202530. doi: 10.1371/journal.pone.0202530 (PMC6095600; doi:10.1371/journal.pone.0202530)
Supplement: S3 Table — (PDF) [file pone.0202530.s003.pdf]

S3 Table

Uniformly detected proteins in 97 CSF samples of MS by targeted quantitative proteomics

Protein	Peptide sequence	Function
kallikrein-6	AVIHPDYDAASHDQDIMLLR<br>ESSQEQSSVVR<br>KPNLQVFLGK<br>LSELIQPLPLER	serine protease, myelination, axon outgrowth
haptoglobin	DIAPTLTLYVGK<br>LRTEGDGVYTLNNEK<br>VTSIQDWVQK<br>YVMLPVADQDQCIR	antibacterial, antioxidant, acute phase response
TIMP-1	EPGLCTWQSLR<br>GFQALGDAADIR	metalloproteinase inhibitor<br>OPC differentiation
apolipoprotein C-II	ESLSSYWESAK<br>TAAQNLYEK<br>TYLPAVDEK	lipoprotein metabolism: VLDL, chylomicron<br>activates lipoprotein lipase
heparin cofactor 2	TLEAQLTPR	thrombin inhibitor; chemotactic for monocytes
gelsolin	AVEVLPK<br>DSQEEEKTEALTSAK<br>EVQGFESATFLGYFK<br>QTQVSVLPEGGETPLFK	calcium-regulated actin-modulating protein
tyrosine-protein kinase receptor UFO (Axl)	APLQGTLLGYR<br>TATITVLPQQPR	binding growth factor GAS6: anti-apoptotic<br>CML oncogene, cell proliferation
lysozyme C	WESGYNTR	enhance the activity of immunoagents, bacteriolytic
beta-2-microglobulin	VEHSDLSFSK<br>VNHVTLSQPK	MHC I
contactin-2	FAQLNLAAEDTR	organization of axonal

	domains at nodes of Ranvier

OPC: oligodendrocyte precursor cell; VLDL: very low-density lipoprotein; CML: chronic myeloid leukemia; GAS6: Growth Arrest Specific 6
