# Supplementary material for: Orthologous proteins of experimental de- and remyelination are differentially regulated in the CSF proteome of multiple sclerosis subtypes
Source: PLoS One. 2018 Aug 16;13(8):e0202530. doi: 10.1371/journal.pone.0202530 (PMC6095600; doi:10.1371/journal.pone.0202530)
Supplement: S4 Table — (PDF) [file pone.0202530.s004.pdf]

S4 Table

Level of peptides relative to the respective stable isotope standard in the CSF of patients with primary progressive (PP), secondary progressive (SP) and relapsing-remitting (RR) multiple sclerosis

APOC2, TAAQNLYEK			UFO, APLQGTLLGYR			TIMP-1, GFQALGDAADIR			B2M, VNHVTLSQPK		
PP	SP	RR	PP	SP	RR	PP	SP	RR	PP	SP	RR
0,077267	0,0788	0,0306	0,0012	0,024619	0,0276	0,031	0,0144	0,0282	0,5764	1,2229	0,402
0,05725	0,04645	0,04425	0,015633	0,01105	0,0153	0,04035	0,0353	0,0141	0,6429	0,5624	0,89515
0,04565	0,034125	0,01355	0,0113	0,0155	0,028667	0,02535	0,0197	0,0326	0,6315	0,5909	1,097475
0,0563	0,0652	0,0423	0,0209	0,0224	0,01165	0,0161	0,035225	0,0229	0,20695	0,5867	0,73895
0,53615	0,05385	0,03815	0,0164	0,01	0,0124	0,0107	0,0495	0,0249	0,2141	0,42265	0,706
0,28825	0,1023	0,047667	0,013	0,019	0,0389	0,03215	0,022633	0,03875	0,4041	0,2819	0,712911
0,136833	0,0577	0,0372	0,01475	0,0052	0,0466	0,0022	0,027	0,0485	0,519067	0,50725	0,607925
0,0642	0,04245	0,01675	0,0037	0,028267	0,00745	0,027133	0,0315	0,0616	0,322033	0,51815	0,437675
0,1681	0,0978	0,0409	0,009333	0,0288	0,016	0,01195	0,08465	0,032925	0,746533	0,1797	0,623
0,028933	0,0172	0,0491	0,0259	0,0131	0,025	0,0159	0,031975	0,0318	0,629633	0,116	0,7932
0,0379	0,0704	0,0413	0,013467	0,01925	0,013	0,019067	0,05275	0,0225	0,6565	0,482067	0,8309
0,032733	0,369233	0,0452	0,0165	0,00755	0,0141	0,023	0,02885	0,01865	0,439733	0,3705	0,5083
0,06445	1,040833	0,0347	0,0049	0,0125	0,03165	0,029333	0,0094	0,01075	0,4618	0,490667	0,3753
0,04235	0,02595	0,0277	0,0086	0,008467	0,020025	0,008	0,071857	0,054	0,3719	0,5552	0,3899
0,0564	0,48865	0,0321	0,0096	0,0004	0,0056	0,026933	0,0933	0,0375	0,9103	0,8005	0,4212
0,06285	0,0423	0,0659	0,007367	0,00665	0,0239	0,0133	0,00135	0,0309	0,64595	0,51735	0,9869
0,04525	0,070095	0,0658	0,02025	0,0208	0,0277	0,0063	0,0329	0,01645	0,328	0,996619	0,4654
0,03975	0,037251	0,0448	0,0081	0,0175	0,03055	0,04265	0,03005	0,039467	0,093	0,781853	0,9114
0,2347	0,0587	0,0314	0,015167	0,0114	0,014825	0,0234	0,0244	0,08	0,3477	0,7075	0,349
0,0511	0,0942	0,0407	0,01285	0,00755	0,010925	0,03655	0,0251	0,0346	0,3224	0,381	0,44145
0,0272	0,0416	0,0496	0,008	0,0011	0,03465	0,036	0,00375	0,0701	0,5349	0,5862	0,47105
0,0351	0,0716	0,042	0,0272	0,007667	0,0257	0,00925	0,0102	0,0541	0,07145	0,594	0,5253
0,0153	0,0432	0,0304	0,0093	0,025136	0,007875	0,0286	0,0177	0,04345	0,4421	0,9314	0,6281
0,0371	0,01825	0,0508	0,0074	0,012467	0,00975	0,012467	0,03225	0,0596	0,6352	0,09385	0,5745
0,0734	0,0484	0,0361	0,01445	0,0108	0,0162	0,0203	0,055299	0,0262	0,5471	0,7502	0,7876
0,040267	0,050667	0,0312	0,0139	0,0163	0,02445	0,0235	0,0191	0,032533	0,42	0,725467	0,57635
0,02665	0,1264	0,0498	0,011267	0,0151	0,0165	0,0204	0,039067	0,0102	0,5269	0,2195	0,6835

0,0357		0,0192	0,01525		0,03095	0,0329		0,0181	0,57985		0,88615
0,0437		0,0235	0,0144		0,024444	0,03965		0,0278	0,5762		0,72865
0,068		0,0488	0,01265		0,0081	0,0272		0,0294	0,4861		0,7748
		0,0509			0,0145			0,0366			0,7083
		0,0876			0,01995			0,04305			0,79605
		0,04075			0,03995			0,02825			0,927
		0,0491			0,0133			0,03275			0,5986
		0,1201			0,0325			0,0221			0,4357
		0,034267			0,01245			0,0696			0,855067
		0,04785			0,0121			0,03915			0,31475
		0,0377			0,0117			0,02665			0,68345
		0,0391			0,0147			0,02015			0,3208
		0,0749			0,00595			0,01295			0,20865
